# External validation and adaptation of the Computational Barthel Index Tool in a large nursing home cohort

**DOI:** 10.1371/journal.pdig.0001793

**Published:** 2026-10-23

**Authors:** Lemba Priscille Ngana, Janusz Wojtusiak, Atefehsadat Haghighathoseini, Bhumi Patel, Dharmi Desai

**Affiliations:** Department of Health Administration, Policy and Informatics, George Mason University, Fairfax, Virginia, United States of America; Instituto Politécnico Nacional Escuela Superior de Medicina: Instituto Politecnico Nacional Escuela Superior de Medicina, MEXICO

## Abstract

The Computational Barthel Index Tool (CBIT), originally developed using U.S. Department of Veterans Affairs (VA) data, predicts future dependency in activities of daily living up to one year in advance. This study externally validated CBIT in a large non-VA nursing home population and evaluated whether population-specific adaptation could recover performance following external transport. We analyzed SEER-Medicare linked data, including 843,836 patients and over 8 million Minimum Data Set (MDS) assessments. The predictor and outcome structures of the original CBIT models were reconstructed in the SEER-Medicare cohort. Pretrained models were evaluated without modification for discrimination and calibration at current, 3-, 6-, and 12-month prediction timeframes. Population-specific adaptation was evaluated using progressively larger subsets of SEER-Medicare training data and compared with model reconstruction in the SEER-Medicare. Under strict external validation, discrimination declined relative to the original VA models, particularly for longer-term predictions. For evaluation models, mean AUC decreased from 0.795 in the original models to 0.693 at current prediction, 0.595 at 3 months, 0.595 at 6 months, and 0.592 at 12 months. Re-evaluation models showed stronger but similarly declining external performance. Calibration error increased, particularly for evaluation models at longer timeframes. Subgroup analyses showed modest variation in discrimination across demographic and clinical groups. Population-specific adaptation substantially recovered discrimination, with performance improving as more SEER-Medicare training data were incorporated. Using the adaptation training set, adapted models achieved performance comparable to SEER-Medicare model reconstruction. External validation revealed substantial performance degradation when CBIT was transported to a non-VA population. Population-specific adaptation recovered much of this performance loss, supporting a structured approach to clinical ML transportability in which pretrained models are externally validated first, followed by adaptation when performance degradation is observed. Full model reconstruction remains an alternative when sufficient local data are available.

## Author summary

Machine learning models are increasingly used in healthcare to help predict patient outcomes and support clinical decision-making. However, models developed in one healthcare system may not perform as well when applied to different patient populations. Understanding how performance changes when a model is evaluated in a new population, and whether performance can be recovered without fully redeveloping the model, is important for successful implementation of clinical machine learning. We evaluated the Computational Barthel Index Tool (CBIT), a model developed using U.S. Department of Veterans Affairs (VA) data to predict future dependency in activities of daily living. We tested the model in a non-VA nursing home population of more than 800,000 patients, including millions of clinical assessments. When applied without modification, CBIT performance declined, particularly for longer-term predictions. We then adapted the model using data from the new population and examined how performance changed as increasing amounts of local data were used. Adaptation substantially recovered performance, with greater improvements observed as more local data were incorporated. We also compared adapted models with models reconstructed using local data. These findings suggest that adapting an existing model may provide a practical alternative to full model redevelopment when local data or development resources are limited.

## Introduction

The Computational Barthel Index Tool (CBIT) is a machine learning (ML)-based model originally developed using data from the U.S. Department of Veterans Affairs (VA) to forecast dependency in Activities of Daily Living (ADLs) up to one year in advance [1]. By leveraging routinely collected structured data - including diagnostic codes, demographic characteristics, and baseline functional status – CBIT enables early identification of individuals at risk for functional decline. Such predictions can inform rehabilitation planning, long-term care decisions, and transitions of care. As populations age and functional disability becomes increasingly prevalent [2], predictive tools that support quality-of-life management are becoming increasingly important for older adults. Maintaining functional independence is particularly important for individuals with serious illness, including cancer [3].

Despite promising internal performance [1], machine learning models frequently fail to generalize beyond the environments in which they are developed. Models that perform well during internal validation may experience substantial performance degradation when applied to new populations due to overfitting, data leakage, or distributional shifts in patient characteristics, coding practices, and healthcare delivery [4–6]. Internal validation approaches, such as cross-validation, bootstrapping, and split-sample testing, estimate performance within the development dataset but cannot substitute for external validation, which evaluates model robustness in independent real-world settings. For this reason, external validation is widely recognized as a critical step before deploying predictive models across healthcare systems [7].

To evaluate CBIT’s transportability, we conducted an external validation using SEER-Medicare linked data, which combine Medicare claims and Minimum Data Set (MDS) assessment data, with cancer information obtained through linkage to the Surveillance, Epidemiology, and End Results (SEER) cancer registry. The MDS is a standardized clinical assessment used for residents of Medicare- and Medicaid-certified nursing homes and provides detailed information on functional status [8]. Together, these data provide structured diagnostic, demographic, and functional status information that enabled reconstruction of the predictor and outcome structure used by the original CBIT models, allowing a rigorous assessment of model performance in a large, non-VA nursing home population.

External validation is particularly important when a model is applied to populations with substantial clinical heterogeneity. We further examined model performance among individuals with cancer. Adults with cancer frequently experience persistent functional limitations resulting from both disease progression and treatment-related effects [9]. Prior studies estimate that approximately one-third to one-half of adults with cancer experience difficulty performing basic or instrumental ADLs [9]. Evaluating CBIT performance separately among cancer and non-cancer populations, therefore, provides insight into whether the model retains discriminatory performance in patients with cancer, who may have different patterns of functional impairment and clinical complexity.

In addition to external validation, we address the commonly observed performance degradation of machine learning models in new populations. When external validation demonstrates performance degradation, an important practical question is whether the original model should be discarded and redeveloped or whether its predictive information can be adapted to a new population. Model updating and adaptation may improve predictive performance while requiring substantially less local data than full model redevelopment, making them particularly attractive for healthcare organizations with limited datasets [10]. The amount of local data available is an important practical consideration: organizations with extensive data resources may be able to develop population-specific models, whereas organizations with more limited local data may prefer to adapt an existing model. However, the amount of external-domain data required for meaningful performance recovery is context-dependent and may vary according to outcome prevalence, population differences, predictor availability, and the adaptation method used.

The objectives of this study were threefold: (1) to reconstruct the predictor and outcome structure of the original CBIT framework within the external nursing home cohort and apply the pretrained CBIT models without retraining or recalibration to establish their performance under strict external validation; (2) to evaluate discrimination, calibration, and variation in model performance across demographic and clinical subgroups, including cancer status and cancer type; and (3) to evaluate population-specific model adaptation using progressively larger subsets of the external nursing home data, characterize how predictive performance changes as the amount of external-domain data available for adaptation increases, and compare the discrimination of adapted and fully redeveloped models.

Together, these analyses address a practical sequence for transporting clinical ML models across healthcare populations: a model should first be externally validated in the target population; if performance is degraded, adaptation using appropriately separated local data can be evaluated; and the amount of local data required for meaningful performance recovery can be empirically characterized. Full model redevelopment provides an alternative when sufficient data and resources are available, and comparison with adaptation can help inform this choice. By evaluating this sequence within a large independent nursing home population, this study provides evidence relevant to the transport, adaptation, and redevelopment of pretrained clinical prediction models across healthcare settings.

## Results

### A. Cohort characteristics

The final external nursing home SEER-Medicare validation cohort included 843,836 patients contributing 8,257,141 MDS evaluations. Patient and assessment characteristics are summarized in Table 1. Compared to the original VA cohort (ORG) used in CBIT development, the SEER-Medicare population was older and had a substantially different demographic composition, particularly with respect to gender and race. The SEER-Medicare cohort was predominantly female, whereas the ORG cohort was predominantly male. The racial distribution also differed between cohorts, with a greater proportion of White patients and a smaller proportion classified as Other in the SEER-Medicare population.

**Table 1:** Sample characteristics of data derived from the original and external seer-medicare cohorts.

	ORG		SEER-Medicare	
	*MDS records*	*Patients*	*MDS records*	*Patients*
	(n = 855,731)	(n = 181,213)	(n = 8,257,141)	(n = 843,836)
**Age, year (mean, SD)**	71.89 ± 12.38	-	79.68 ± 10.62	-
**Gender (%)**				
**Male**	96.8	96.9	37.0	40.3
**Female**	3.2	3.1	63.0	59.7
**Race (%)**				
**White**	58.8	55.4	78.5	79.4
**Black**	13.0	11.9	12.8	10.7
**Asian**	1.5	1.4	3.3	3.9
**Other**	26.7	31.3	5.4	6.0
**Non-cancer (%)**	-	-	45.2	37.5
**Cancer (%)**			54.8	62.5
**Breast**	-	-	17.4	17.3
**Lung**	-	-	17.4	22.4
**Pancreas**	-	-	3.1	4.3
**Prostate**	-	-	11.6	13.0
**Stomach**	-	-	2.2	2.5
**Other**	-	-	3.1	3
**>1 cancer**	-	-	0.9	1.3
**CCS^max^ (mean, SD)**	1424.65 ± .1215.70	-	1101.08 ± 1034.03	-
**CCS^min^ (mean, SD)**	619.75 ± 867.49	-	491.94 ± 706.37	-
**Barthel Score (mean, SD)**	49.00 ± 29.98	-	33.15 ± 23.93	-

Abbreviations: MDS = Minimum Dataset; ORG = data from original paper [1]

Functional dependency was highly prevalent in the SEER-Medicare cohort (Table 2). Across the nine ADL domains, dependency was particularly common for bathing, dressing, grooming, toileting, transferring, and walking. The lower mean Barthel Score observed in the external cohort (33.15 vs. 49.00 in the ORG cohort) was consistent with greater overall functional dependence in the external validation population.

**Table 2:** Functional dependency in the original (ORG) and external SEER-medicare cohorts.

	ORG		SEER-Medicare	
	*MDS records*	*Patients*	*MDS records*	*Patients*
	(n = 855,731)	(n = 181,213)	(n = 8,257,141)	(n = 843,836)
**Any level of dependency (%)**				
**Bathing**	74.2	77.5	67.4	94.8
**Bladder Control**	39.7	43	40.2	62.3
**Bowel Incontinence**	41.4	45.3	32.5	54.7
**Dressing**	66.2	71.4	63.4	92.9
**Eating**	47.8	54.6	48.1	76.8
**Grooming**	63	67.1	59.7	88.3
**Toileting**	60.9	66.8	64.4	92.9
**Transferring**	52.1	60.9	61.8	92.3
**Walking**	73.2	80.1	64.6	94.6

Cancer was also common in the SEER-Medicare population. Among patients, 62.5% had at least one cancer diagnosis represented in the study, with lung and breast cancer being the most prevalent individual cancer types. A smaller proportion of patients had multiple cancer types.

Overall, the external validation cohort differed substantially from the original development population in demographic characteristics and functional status. These differences provide important context for interpreting the transportability of the pretrained CBIT models to a heterogeneous nursing home population outside the VA healthcare system.

### B. Overall performance

The pretrained CBIT models were applied directly to the external validation cohort without retraining, recalibration, or modification of model parameters. Performance across the four prediction timepoints and for both evaluation models (*M*_*E*_^*d*^_*τ*_) and re-evaluation models (*M*_*RE*_^*d*^) is summarized in Table 3.

**Table 3:** Mean ± standard deviation of model performance metrics in the original (ORG) and External SEER-medicare cohorts across prediction timeframes.

	ORG				SEER-Medicare			
	Accuracy	AUC	Precision	Recall	Accuracy	AUC	Precision	Recall
**Evaluation Models M_E_^d^_τ_**								
**Current**	.743 ± .029	.795 ± .010	.743 ± .046	.800 ± .128	.667 ± .109	.693 ± .030	.861 ± .101	.624 ± .180
**3 Months**	.727 ± .037	.761 ± .006	.734 ± .049	.783 ± .161	.625 ± .125	.595 ± .035	.865 ± .089	.559 ± .200
**6 Months**	.720 ± .038	.746 ± .009	.721 ± .045	.729 ± .238	.525 ± .141	.595 ± .032	.881 ± .084	.410 ± .215
**12 Months**	.716 ± .039	.725 ± .016	.696 ± .073	.701 ± .264	.488 ± .155	.592 ± .034	.892 ± .068	.344 ± .246
**Re-evaluation Models M_RE_^d^**								
**Current**	.900 ± .007	.947 ± .006	.910 ± .011	.907 ± .041	.818 ± .025	.940 ± .013	.870 ± .025	.900 ± .034
**3 Months**	.815 ± .020	.876 ± .011	.849 ± .019	.816 ± .094	.731 ± .025	.738 ± .011	.800 ± .025	.810 ± .034
**6 Months**	.759 ± .029	.808 ± .014	.784 ± .029	.737 ± .165	.727 ± .027	.742 ± .012	.731 ± .025	.726 ± .041
**12 Months**	.737 ± .035	.772 ± .022	.742 ± .049	.699 ± .226	.720 ± .028	.735 ± .011	.727 ± .025	.670 ± .053

**Note:** Mean ± SD represents the mean and standard deviation of performance estimates across the nine ADL outcomes for each model type and prediction timeframe.

Overall, model discrimination was lower in the external SEER-Medicare population than in the original VA cohort, with the magnitude of the decline varying by model type and prediction timeframe. The evaluation models showed the greatest degradation, with AUCs declining to approximately 0.59 at 3, 6, and 12 months, indicating discrimination only modestly above chance for longer-term predictions. In contrast, the re-evaluation models consistently demonstrated higher discrimination than the evaluation models, especially for current-status prediction, although their performance also declined as the prediction timeframe increased.

The difference between evaluation and re-evaluation models was most pronounced for current-status prediction, where incorporation of a previous functional assessment corresponded to higher discrimination in the external population. For both model types, however, discrimination generally decreased for the 3-, 6-, and 12-month prediction timeframes, indicating increasing difficulty in predicting functional dependency as the prediction time increased. The decline was particularly pronounced for the evaluation models, for which discrimination at 3–12 months was only modestly above chance.

Accuracy, precision, and recall showed a similar distinction between model types. Evaluation models generally demonstrated higher precision than recall, indicating that predictions classified as dependency were often correct but that a proportion of patients with observed dependency were not identified at the prespecified probability threshold. Re-evaluation models showed higher and more balanced precision and recall, particularly for shorter prediction timeframes.

Collectively, the external validation results indicate that the original CBIT did not maintain their full level of discrimination when applied to the external SEER-Medicare population without adaptation. Performance degradation was particularly evident for the evaluation models, for which discrimination at longer prediction timepoints approached chance levels. In contrast, the re-evaluation models retained stronger discrimination, although performance also declined with increasing prediction time. These findings indicate that transportability varied by model type and prediction timeframe and provide a basis for the subsequent calibration and model adaptation analyses. Detailed ADL-specific performance results are provided in S1 Table.

### C. Subgroup performance

Subgroup analyses were conducted to examine variation in model discrimination across demographic and clinical characteristics, including sex, race, age, cancer status, and cancer type (Tables 4–8). Across subgroups, the general pattern observed in the overall external validation analysis was maintained, with discrimination generally decreasing as the prediction timeframe increased. Between-subgroup differences in AUC were generally modest relative to differences associated with model type and prediction timeframe, although the magnitude and direction of these differences varied across model type, prediction timeframe, and subgroup.

**Table 4:** AUC ± standard deviation in the external SEER-medicare cohort across timepoints, by sex.

	SEER-Medicare		*p-value summary*
	*Male*	*Female*	
**Evaluation Models M_E_^d^_τ_**			
**Current**	.695 ± .025	.687 ± .037	some ADLs *p* < 0.05
**3 Months**	.589 ± .026	.596 ± .037	some ADLs *p* < 0.05
**6 Months**	.586 ± .030	.598 ± .038	some ADLs *p* < 0.05
**12 Months**	.582 ± .031	.595 ± .039	some ADLs *p* < 0.05
**Re-evaluation Models M_RE_^d^**			
**Current**	.941 ± .015	.933 ± .012	some ADLs *p* < 0.05
**3 Months**	.737 ± .013	.744 ± .018	some ADLs *p* < 0.05
**6 Months**	.739 ± .016	.751 ± .013	some ADLs *p* < 0.05
**12 Months**	.734 ± .012	.747 ± .015	some ADLs *p* < 0.05

**Table 5:** AUC ± standard deviation in the external SEER-medicare cohort across timepoints, by race.

	SEER-Medicare				*p-value summary*
	*White*	*Black*	*Asian*	*Other*	
**Evaluation Models M_E_^d^_τ_**					
**Current**	.687 ± .036	.718 ± .032	.709 ± .036	.711 ± .030	all ADLs *p* < 0.05
**3 Months**	.595 ± .037	.594 ± .033	.586 ± .036	.591 ± .028	all ADLs *p* < 0.05
**6 Months**	.595 ± .036	.596 ± .034	.586 ± .022	.593 ± .032	all ADLs *p* < 0.05
**12 Months**	.592 ± .027	.595 ± .028	.582 ± .035	.592 ± .039	all ADLs *p* < 0.05
**Re-evaluation Models M_RE_^d^**					
**Current**	.937 ± .012	.955 ± .014	.953 ± .011	.952 ± .014	all ADLs *p* < 0.05
**3 Months**	.743 ± .010	.723 ± .013	.701 ± .013	.718 ± .012	all ADLs *p* < 0.05
**6 Months**	.746 ± .017	.728 ± .014	.708 ± .015	.724 ± .017	all ADLs *p* < 0.05
**12 Months**	.738 ± .010	.725 ± .011	.705 ± .018	.719 ± .009	all ADLs *p* < 0.05

**Table 6:** AUC ± standard deviation in the external SEER-medicare cohort across timepoints, by age group.

	SEER-Medicare						*p-value summary*
	*<60*	*60-64*	*65-69*	*70-74*	*75-79*	*>=80*	
**Evaluation Models M_E_^d^_τ_**							
**Current**	.711 ± .046	.675 ± .032	.661 ± .037	.658 ± .043	.663 ± .038	.673 ± .029	all ADLs *p* < 0.05
**3 Months**	.631 ± .037	.603 ± .033	.587 ± .036	.582 ± .033	.582 ± .028	.582 ± .031	all ADLs *p* < 0.05
**6 Months**	.629 ± .043	.600 ± .034	.584 ± .036	.580 ± .043	.578 ± .048	.582 ± .040	all ADLs *p* < 0.05
**12 Months**	.622 ± .048	.597 ± .028	.580 ± .035	.577 ± .042	.576 ± .042	.581 ± .043	all ADLs *p* < 0.05
**Re-evaluation Models M_RE_^d^**							
**Current**	.954 ± .010	.943 ± .013	.935 ± .010	.932 ± .015	.933 ± .011	.939 ± .014	all ADLs *p* < 0.05
**3 Months**	.809 ± .012	.783 ± .013	.761 ± .009	.749 ± .015	.740 ± .019	.718 ± .015	all ADLs *p* < 0.05
**6 Months**	.812 ± .011	.784 ± .014	.765 ± .015	.753 ± .021	.742 ± .015	.723 ± .017	all ADLs *p* < 0.05
**12 Months**	.808 ± .012	.782 ± .012	.762 ± .017	.750 ± .021	.738 ± .014	.717 ± .015	all ADLs *p* < 0.05

**Table 7:** AUC ± standard deviation in the external SEER-medicare cohort across timepoints, by cancer status.

	SEER-Medicare		*p-value summary*
	*Non-cancer*	*Cancer*	
**Evaluation Models M_E_^d^_τ_**			
**Current**	.710 ± .036	.672 ± .038	all ADLs *p* < 0.05
**3 Months**	.598 ± .038	.586 ± .037	all ADLs *p* < 0.05
**6 Months**	.595 ± .029	.580 ± .032	all ADLs *p* < 0.05
**12 Months**	.599 ± .036	.578 ± .040	all ADLs *p* < 0.05
**Re-evaluation Models M_RE_^d^**			
**Current**	.949 ± .013	.931 ± .014	all ADLs *p* < 0.05
**3 Months**	.734 ± .013	.735 ± .015	all ADLs *p* < 0.05
**6 Months**	.730 ± .015	.732 ± .014	all ADLs *p* < 0.05
**12 Months**	.735 ± .014	.724 ± .018	all ADLs *p* < 0.05

**Table 8:** AUC ± standard deviation in the external SEER-medicare cohort across timepoints, by cancer type.

	SEER-Medicare					*p-value summary*
	*Breast*	*Lung*	*Pancreas*	*Prostate*	*Stomach*	
**Evaluation Models M_E_^d^_τ_**						
**Current**	.679 ± .037	.654 ± .036	.656 ± .036	.679 ± .035	.673 ± .037	all ADLs *p* < 0.05
**3 Months**	.594 ± .038	.571 ± .036	.573 ± .033	.581 ± .030	.581 ± .027	all ADLs *p* < 0.05
**6 Months**	.588 ± .037	.566 ± .029	.568 ± .021	.574 ± .032	.584 ± .034	all ADLs *p* < 0.05
**12 Months**	.586 ± .049	.563 ± .015	.564 ± .036	.572 ± .042	.569 ± .041	all ADLs *p* < 0.05
**Re-evaluation Models M_RE_^d^**						
**Current**	.931 ± .013	.926 ± .014	.925 ± .014	.934 ± .016	.936 ± .012	all ADLs *p* < 0.05
**3 Months**	.739 ± .014	.730 ± .014	.723 ± .014	.730 ± .010	.722 ± .009	all ADLs *p* < 0.05
**6 Months**	.734 ± .013	.728 ± .016	.722 ± .020	.727 ± .014	.717 ± .009	all ADLs *p* < 0.05
**12 Months**	.725 ± .018	.719 ± .011	.715 ± .013	.723 ± .015	.707 ± .016	all ADLs *p* < 0.05

Because these subgroup analyses were based on AUC, they were used to describe variation in model discrimination rather than to establish algorithmic fairness, clinical equivalence, or the absence of disparities. Statistical significance was therefore interpreted together with the magnitude of the observed AUC differences, particularly given the large size of the external cohort.

#### 1. Sex differences.

Model discrimination was broadly similar between male and female patients across prediction timeframes for both evaluation (*M*_*E*_^*d*^_*τ*_) and re-evaluation (*M*_*RE*_^*d*^) models (Table 4). Differences between the two groups were relatively small compared with the overall differences between evaluation and re-evaluation models. As observed in the overall analysis, discrimination decreased with increasing prediction timeframe for both groups. Although some individual ADL comparisons reached statistical significance, the magnitude of the between-sex AUC differences was generally small.

#### 2. Race differences.

AUC varied across racial groups for both evaluation and re-evaluation models (Table 5). The direction and magnitude of these differences varied by prediction timeframe and model type, while the overall decline in discrimination with increasing prediction timeframe was observed across all racial groups. Statistical testing indicated significant differences in AUC across racial groups for all ADLs (*p* < 0.05); however, the magnitude of these differences was generally modest. For example, among evaluation models, current-status AUC ranged from 0.687 to 0.718 across racial groups, while AUC at 3–12 months differed only modestly across groups. The re-evaluation models showed somewhat greater variation at the longer prediction timepoints, particularly for Asian patients, but the overall pattern of declining discrimination with increasing prediction timeframe remained consistent. Given the large size of the external SEER-Medicare cohort, statistically significant differences should therefore be interpreted in the context of the magnitude of the observed AUC differences rather than as evidence of clinically meaningful disparities in model discrimination.

#### 3. Age group differences.

Model discrimination varied across age groups (Table 6), with higher AUCs generally observed among younger patients and lower AUCs among older age groups, particularly for the re-evaluation models at longer prediction timeframes. This age-related pattern was most pronounced for the re-evaluation models, for which AUC decreased progressively across age groups from <60 years to ≥80 years at 3, 6, and 12 months. For example, at 12 months, AUC decreased from 0.808 among patients younger than 60 years to 0.717 among patients aged ≥80 years. Evaluation models showed a similar, although less consistent, pattern, with the highest AUCs generally observed among patients younger than 60 years, while AUCs among the older age groups were relatively similar. The decline in discrimination with increasing prediction timeframe was observed within each age group for both model types. Statistical testing indicated significant differences in AUCs across age groups for all ADLs (*p* < 0.05); however, the magnitude of these differences varied by model type and prediction timeframe.

#### 4. Cancer status differences.

Model discrimination differed by cancer status (Table 7). Evaluation models generally showed higher discrimination among non-cancer patients than among cancer patients, with the largest differences observed for current-status prediction and smaller differences at longer prediction timeframes. For example, current-status AUC was 0.710 among non-cancer patients compared with 0.672 among cancer patients, while at 12 months, the corresponding AUCs were 0.599 and 0.578, respectively. In contrast, discrimination for the re-evaluation models was generally similar between cancer and non-cancer groups, particularly at 3 and 6 months, although a larger difference was observed for current-status prediction (0.949 vs. 0.931). Statistical testing indicated significant differences in AUC across cancer-status groups for all ADLs (*p* < 0.05); however, the magnitude of the observed differences varied by model type and prediction timeframe.

#### 5. Cancer type differences.

Among patients with cancer, model discrimination varied across cancer types (Table 8). For the evaluation models, breast and prostate cancer generally showed higher AUCs than lung and pancreatic cancer, particularly for current-status prediction, although the magnitude of these differences was modest. For example, current-status AUC ranged from 0.654 for lung cancer to 0.679 for breast and prostate cancer. At longer prediction timeframes, AUCs were more similar across cancer types, although some variation remained. Differences among cancer types were generally smaller for the re-evaluation models, although some variation remained across prediction timeframes. Discrimination generally decreased with increasing prediction timeframe across cancer types for both model types. Statistical testing indicated significant differences in AUC across cancer types for all ADLs (*p* < 0.05); however, the magnitude of the between-group differences was generally modest.

Overall, subgroup analyses demonstrated some variation in discrimination across demographic and clinical characteristics. The most consistent patterns were associated with prediction timeframe and model type, while the magnitude and direction of subgroup differences varied across characteristics. These findings provide a descriptive assessment of variation in model discrimination within the external SEER-Medicare cohort; interpretation of subgroup performance is limited to discrimination and does not constitute a comprehensive assessment of algorithmic fairness or clinical equivalence.

### D. Calibration

Calibration was evaluated using calibration curves complemented by expected calibration error (ECE) and Brier scores across the current, 3-, 6-, and 12-month prediction timeframes (Table 9). Calibration performance differed substantially between the evaluation (*M*_*E*_^*d*^_*τ*_) and re-evaluation (*M*_*RE*_^*d*^) models.

**Table 9:** Mean ECE and brier score in the external SEER-medicare cohort across prediction timeframes.

	SEER-Medicare	
	*ECE*	*Brier Score*
**Evaluation Models M_E_^d^_τ_**		
**Current**	0.233	0.208
**3 Months**	0.257	0.223
**6 Months**	0.335	0.266
**12 Months**	0.363	0.282
**Re-evaluation Models M_RE_^d^**		
**Current**	0.033	0.059
**3 Months**	0.058	0.064
**6 Months**	0.092	0.071
**12 Months**	0.128	0.082

The evaluation models showed higher calibration error in the external SEER-Medicare cohort, with ECE increasing from 0.233 for current-status prediction to 0.363 at 12 months. Brier scores showed a similar pattern, increasing from 0.208 to 0.282 across the same prediction timeframes. These results indicate worsening probabilistic performance as the prediction timeframe increased. However, visual inspection of the calibration curves showed that predicted and observed probabilities generally remained reasonably close to the diagonal reference line, despite the increases in ECE and Brier score.

In contrast, the re-evaluation models showed substantially lower calibration error across all prediction timeframes. ECE increased from 0.033 for current status prediction to 0.128 at 12 months, while Brier scores increased from 0.059 to 0.082. Thus, although calibration deteriorated with increasing prediction timeframe, the magnitude of this deterioration was smaller than that observed for the evaluation models. The calibration curve for bathing (Fig 1) illustrated the comparatively better calibration of the re-evaluation models.

**Fig 1:**
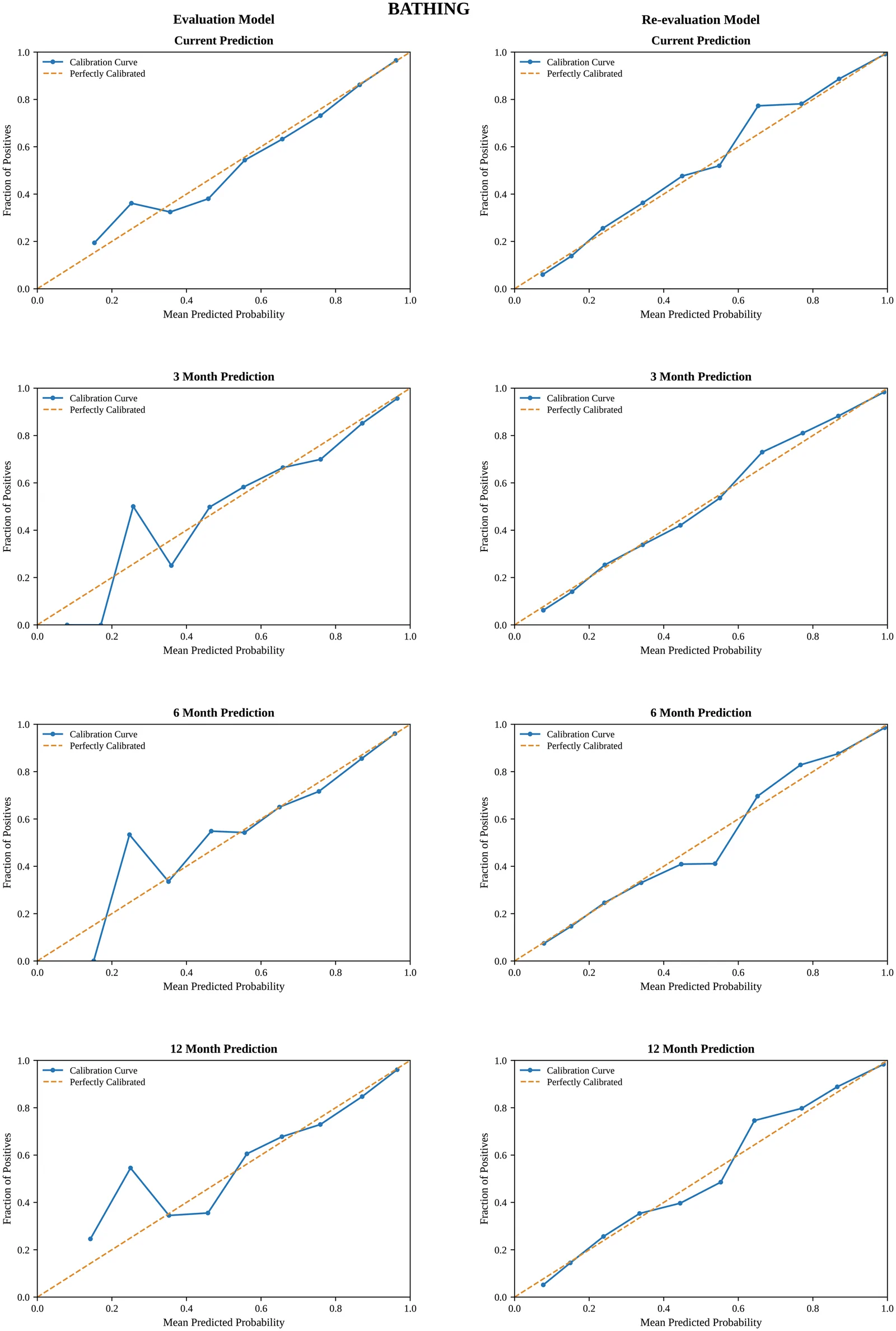
Evaluation Model (*M*_*E*_^*d*^_*τ*_) and Re-evaluation Model (*M*_*RE*_^*d*^) calibration plots for the Bathing outcome.

Overall, the calibration results indicate that external validation affected the probabilistic performance of the pretrained CBIT models, with greater calibration error observed for the evaluation models and at longer prediction timeframes. At the same time, the calibration curves generally showed reasonable agreement between predicted and observed probabilities, suggesting that the numerical calibration-error measures should be interpreted alongside the visual calibration assessments. These findings provide a basis for evaluating whether population-specific model adaptation can further improve predictive performance in the external SEER-Medicare population. Full calibration plots for all nine ADLs across the prediction timeframes are provided in S1 Fig.

### E. Model adaptation

To assess whether population-specific adaptation could recover performance lost during external validation, the pretrained CBIT models were adapted to the external SEER-Medicare population using progressively larger subsets of the external nursing home cohort. Adaptation was evaluated separately from the strict external validation analysis. For the primary adaptation analysis summarized in Table 10, 80% of the available adaptation data were used for model training and 20% were reserved as a held-out patient-level test set. Model performance was evaluated on the held-out test set across the prediction timeframes.

**Table 10:** Comparison of original, SEER-medicare model reconstruction, SEER-medicare external validation, and SEER-medicare model adaptation.

	SEER-Medicare			
	*ORG*	*Model Reconstruction*	*External Validation*	*Model Adaptation*
**Evaluation Models M_E_^d^_τ_**				
**Current**	.795 ± .010	.741 ± .005	.693 ± .030	.743 ± .030
**3 Months**	.761 ± .006	.700 ± .006	.595 ± .035	.703 ± .028
**6 Months**	.746 ± .009	.692 ± .008	.595 ± .032	.693 ± .032
**12 Months**	.725 ± .016	.682 ± .009	.592 ± .034	.683 ± .039
**Re-evaluation Models M_RE_^d^**				
**Current**	.947 ± .006	.961 ± .010	.940 ± .013	.961 ± .014
**3 Months**	.876 ± .011	.960 ± .008	.738 ± .011	.961 ± .012
**6 Months**	.808 ± .014	.960 ± .010	.742 ± .012	.960 ± .017
**12 Months**	.772 ± .022	.960 ± .011	.735 ± .011	.960 ± .009

Adaptation improved discrimination relative to direct application of the pretrained models. For the evaluation models (M_E_^d^_τ_), adaptation increased AUC across all prediction timeframes relative to direct external validation, recovering a substantial portion of the observed performance loss, although AUC remained below the corresponding original-model performance. Improvement was also observed for the re-evaluation models (M_RE_^d^), which achieved consistently high discrimination following adaptation across prediction timeframes. These results indicate that incorporating external domain data during model adaptation reduced the performance degradation observed during direct external validation.

To examine how the amount of external-domain data available for adaptation influenced performance, additional adaptation analyses were conducted using progressively larger proportions of the available training data (Fig 2). Increasing the proportion of adaptation training data was associated with improved discrimination, with gains observed even when relatively small subsets of the available adaptation data were used. Performance gains became smaller at higher proportions of the adaptation dataset, suggesting that the incremental benefit of additional adaptation data may diminish beyond a certain point. These analyses were distinct from the primary 80% training/20% testing analysis presented in Table 10 and were used to characterize the relationship between adaptation dataset size and AUC.

**Fig 2:**
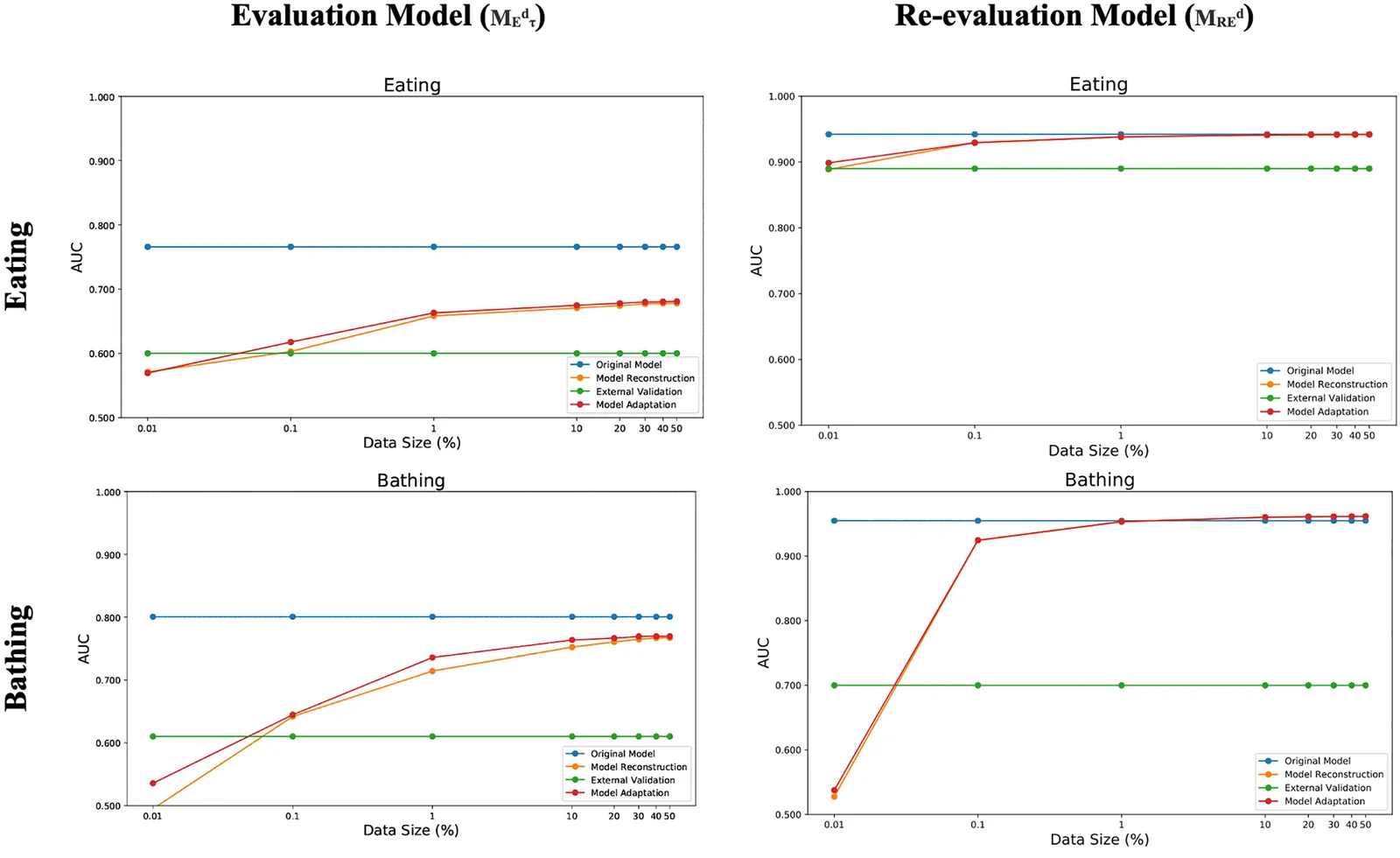
AUC of evaluation (*M*_*E*_^*d*^_*τ*_) and re-evaluation (*M*_*RE*_^*d*^) model adaptation across varying adaptation dataset sizes for eating and bathing.

For the re-evaluation models, adaptation increased AUC to approximately 0.96 across all prediction timeframes, closely matching the corresponding SEER-Medicare model-reconstruction results and substantially exceeding the AUCs observed under strict external validation, particularly for the 3-, 6-, and 12-month prediction timeframes. This pattern indicates that population-specific model development and adaptation substantially improved discrimination for the re-evaluation models relative to external application.

All primary adaptation models reported in Table 10 were evaluated on the held-out patient-level test set that was not used during adaptation. Thus, the AUC values reported in Table 10 reflect performance on patients not included in model training. The progressively sized adaptation analyses shown in Fig 2 similarly evaluated performance on held-out data corresponding to each adaptation training size. These results should be interpreted as evidence of improved discrimination within the evaluated external SEER-Medicare cohort rather than as evidence of clinical superiority or generalizability to other populations.

Overall, the adaptation analysis demonstrated that population-specific adaptation of the pretrained CBIT models improved discrimination in the external cohort compared with direct external application. The results further suggest that performance recovery may be achievable with relatively limited amounts of external-domain data, although the extent of improvement varied by model type and prediction timeframe. Detailed AUC results for individual ADLs across prediction timeframes and adaptation dataset sizes are provided in S2 Fig.

## Discussion

This study externally validated the Computational Barthel Index Tool (CBIT), a machine learning (ML)-based framework originally developed using U.S. Department of Veterans Affairs (VA) data to predict future dependency in activities of daily living (ADLs). Using a large nursing home SEER-Medicare cohort with reconstructed predictor and outcome structures, we evaluated the performance of the pretrained CBIT models in a non-VA nursing home population and subsequently examined whether population-specific adaptation could recover performance. The findings demonstrate three related aspects of model transportability: performance degradation can occur when a pretrained model is applied to a new population, population-specific adaptation can recover a substantial portion of this performance loss, and performance recovery varied with the amount of local data available for adaptation. Comparison with SEER-Medicare reconstruction further provides a basis for considering adaptation versus redevelopment when deploying pretrained models in new healthcare populations.

Under strict external validation, CBIT discrimination declined relative to the original VA performance, particularly at the 3-, 6-, and 12-month prediction timeframes. The degradation was most pronounced for the evaluation models, for which AUCs at longer prediction timepoints were only modestly above chance. In contrast, the re-evaluation models, which incorporate prior functional status, retained substantially stronger discrimination. These findings are consistent with previous research demonstrating that ML prediction models may experience meaningful performance degradation when transported beyond their development populations [11–14]. The comparatively high discrimination of the adapted re-evaluation models may reflect the predictive information contained in prior functional status, which was available to these models and may provide a strong longitudinal indicator of subsequent dependency.

Several factors may explain the observed reduction in external performance. Models developed within a single healthcare system may capture relationships that are partly specific to the development population, potentially limiting transportability [15]. In addition, differences in cohort construction and data pre-processing between the VA and external SEER-Medicare populations may have altered the distribution and clinical meaning of model predictors [16,17]. The external SEER-Medicare cohort represented a nursing home population with different demographic and clinical characteristics from the original VA population, and differences in data structure, coding practices, and clinical measurement between the VA and SEER-Medicare datasets may also have contributed to differences in predictor distributions and outcome ascertainment. These findings reinforce the importance of independent external validation before pretrained clinical prediction models are deployed in substantially different healthcare populations [7,13,15].

The subgroup analyses indicated that the largest and most consistent sources of performance variation were model type and prediction timeframe rather than large differences across demographic or clinical groups**.** Although statistically significant differences in AUC were observed across several subgroups, the absolute differences were generally modest compared with the differences between evaluation and re-evaluation models and across prediction timeframes. Performance generally declined with increasing prediction time across subgroups, while re-evaluation models maintained higher discrimination than evaluation models.

Cancer status and cancer type were examined because cancer patients may experience distinct patterns of functional impairment related to disease progression, treatment effects, comorbidity, and survivorship [9,10]. Evaluation models showed somewhat lower discrimination among cancer patients than non-cancer patients, particularly for current-status prediction, whereas re-evaluation models showed more similar discrimination between the two groups. Among patients with cancer, AUC varied across cancer types, but the magnitude of these differences was generally modest. Thus, although some heterogeneity in discrimination was observed, the magnitude of the differences across cancer-status and cancer-type subgroups was generally modest relative to the differences associated with model type and prediction timeframe. These findings support evaluating model performance in clinically distinct populations while recognizing that differences in AUC alone do not establish algorithmic unfairness, clinically meaningful disparities, or equivalence in clinical performance.

### Model adaptation and the role of local data

An important contribution of this study is that it extended beyond conventional external validation to evaluate whether population-specific adaptation could recover performance. We evaluated whether the CBIT framework could be adapted to the nursing home SEER-Medicare population using local data and examined how performance changed as progressively larger amounts of external SEER-Medicare data became available. Adaptation substantially improved discrimination relative to direct external application. For the evaluation models, adaptation recovered a substantial portion of the performance loss observed during external validation, although performance remained below the original VA model performance. For the re-evaluation models, adaptation increased discrimination across prediction timeframes, comparable to the SEER-Medicare model-reconstruction results.

The data-size analyses further demonstrated that the amount of local data available for adaptation is an important consideration when transporting clinical ML models. Performance generally improved as larger subsets of SEER-Medicare training data were used for adaptation, with smaller incremental gains at higher data proportions. This pattern suggests that the incremental benefit of additional adaptation data may diminish at higher data proportions, although the present analyses do not establish a universal minimum sample size or a threshold at which adaptation becomes sufficient for deployment. Rather, they demonstrate that meaningful performance recovery can occur using substantially less local data than may be required for full model reconstruction.

The comparison between adaptation and SEER-Medicare model reconstruction provides an important practical perspective. When sufficient local data and technical resources are available, full model reconstruction or redevelopment may be appropriate for developing a population-specific model. However, external performance degradation does not necessarily mean that a pretrained model must be discarded and completely redeveloped. When local data are more limited, population-specific adaptation may offer a more practical alternative. The appropriate strategy depends on the extent of external performance degradation, the amount and representativeness of available local data, and the resources available for model development and validation**.**

These findings support a practical sequence for transporting clinical ML models across healthcare settings: first externally validate the pretrained model; if performance is degraded in the target population, evaluate population-specific adaptation using appropriately separated local data; and, when sufficient data and resources are available, compare adaptation with full model reconstruction. Importantly, this study does not establish a universal data threshold for choosing adaptation over redevelopment. Instead, it provides empirical evidence that the relationship between local data availability and performance can be evaluated directly when planning model deployment.

### Implications for clinical ML deployment

The findings have implications for healthcare organizations seeking to implement pretrained ML models across institutions or patient populations. Large healthcare systems with extensive longitudinal data may have sufficient resources to reconstruct or redevelop models for their specific populations. Organizations with more limited datasets may consider adapting an existing model, particularly when the pretrained model retains useful predictive information in the target population.

Adaptation should not, however, be interpreted as a substitute for external validation. The strong performance observed after adaptation was evaluated on a held-out patient-level SEER-Medicare test set and therefore represents performance within the evaluated target population rather than evidence of generalizability to other healthcare systems. Although adaptation improved discrimination, calibration should also be considered when evaluating model suitability for deployment, particularly because calibration error increased at longer prediction timeframes under external validation. Additional external validation would be required before assuming that adapted models would perform similarly in other nursing home populations or healthcare settings. Similarly, the present study evaluated discrimination and calibration but does not establish clinical utility, impact on clinical decision-making, or patient outcomes following implementation.

### Strengths and limitations

This study has several strengths. First, it used a large nursing home SEER-Medicare cohort and reconstructed the predictor and outcome structures of the original CBIT framework, enabling evaluation in an independent non-VA population. Second, the study evaluated multiple prediction timeframes and examined performance across demographic and clinically relevant subgroups, including cancer status and cancer type. Third, the study went beyond conventional external validation by systematically evaluating population-specific adaptation across increasing amounts of local data and comparing adapted models with model reconstruction in the SEER-Medicare.

Several limitations should also be considered. First, the study focused on nursing home residents, which may limit generalizability to younger or community-dwelling populations. In addition, requiring at least three consecutive years of MDS assessments may have preferentially selected long-stay nursing home residents with greater longitudinal observation and excluded shorter-stay residents, patients with rapid clinical deterioration, and those who died or were discharged before completing the observation requirement. Because a less restrictive longitudinal criterion was not evaluated, the extent to which this selection affected model transportability could not be quantified. Second, the cohort also included some Medicare beneficiaries younger than 65 years who qualified for Medicare through disability. Because SEER-Medicare does not necessarily represent the broader population of individuals younger than 65 years with disability, findings for this age group may have limited generalizability. We therefore interpret the results as applying to the restricted SEER-Medicare nursing home population studied rather than to the broader population of younger adults with disability. Third, we did not restrict the cohort to beneficiaries with continuous Part A and Part B coverage and no managed-care enrollment, which are commonly used criteria in SEER-Medicare studies to identify individuals likely to have complete claims data. Consequently, some claims-based diagnoses or healthcare utilization information may have been incompletely captured, potentially introducing misclassification of claims-derived predictors [18]. The extent of this potential bias could not be quantified in the present analysis. Fourth, the SEER-Medicare cohort is limited to cancer patients identified through SEER registry areas and a 5% sample of Medicare beneficiaries without SEER-identified cancer and therefore should not be interpreted as representative of the broader U.S. Medicare or nursing home population. Fifth, reconstruction of the CBIT predictor and outcome structures required harmonization of variables across data sources, and differences in coding systems or measurement practices may have contributed to observed performance differences. Such representation and encoding decisions can influence the performance of clinical ML models, even when the underlying clinical information is similar [19]. Sixth, although the data-size analyses demonstrated a relationship between local adaptation data and AUC, they do not establish a universal sample-size threshold for performance recovery because the amount of data required is likely to depend on outcome prevalence, population characteristics, predictor distributions, and the adaptation method. Finally, improved discrimination following adaptation does not by itself establish clinical superiority, clinical utility, or generalizability beyond the external nursing home SEER-Medicare population. In addition, subgroup analyses were limited primarily to discrimination using AUC and did not include subgroup-specific calibration, error-rate parity, or other formal fairness metrics; therefore, similarity in AUC across subgroups should not be interpreted as evidence of equivalent calibration, error rates, or algorithmic fairness.

## Methods - design of the validation study

### A. Computational Barthel Index Tool (CBIT) development

The Computational Barthel Index Tool (CBIT) is a machine learning-based framework developed by [1] to predict future dependency in activities of daily living (ADLs) among nursing home residents using routinely collected clinical and administrative data from the U.S. Department of Veterans Affairs (VA). Because the present study focuses on external validation rather than original model development, only a brief overview of the published CBIT framework is provided below.

CBIT used routinely collected structured clinical data as predictors. Input features included demographic characteristics (age, race, and sex) and clinical diagnoses. Diagnosis codes recorded using ICD-9-CM were mapped to 281 diagnostic categories using the Clinical Classifications Software (CCS) developed by the Agency for Healthcare Research and Quality (AHRQ).

Two temporal variables summarized each CCS condition. *ccs*_i_^min^ measured the time since the most recent diagnosis, whereas *ccs*_i_^max^ measured the time since the first recorded diagnosis, thereby capturing disease recency and chronicity:

*ccs*_i_^min^_i,j_ = *d*_*prediction,i*_ *– d*_*most recent,i,j*_

*ccs*_i_^max^_i,j_ = *d*_*prediction,i*_ *– d*_*first recorded,i,j*_

where *d*_*prediction,i*_ denotes the prediction date for patient *i*, and *d*_*most recent,i,j*_ and *d*_*first recorded,i,j*_ correspond to the dates of the most recent and first recorded diagnoses of condition *j* for patient *i*.

Model development followed a standard machine learning workflow. The dataset was divided into training (90%) and test (10%) sets. Within the training set, ten-fold cross-validation was used for hyperparameter tuning and model selection.

In total, 72 predictive models were developed in the original CBIT framework [1]. These included two model types: evaluation models (*M*_*E*_^*d*^_*τ*_), designed for patients without prior MDS assessments, and re-evaluation models (*M*_*RE*_^*d*^), designed for patients with previous assessments. Each model predicted dependency across nine ADL domains at four timepoints (current status, 3, 6, and 12 months), resulting in 36 evaluation models and 36 re-evaluation models [1].

In the present study, the original CBIT models were not retrained, recalibrated, or otherwise modified before external validation. The pretrained models were applied directly to the independent external nursing home SEER-Medicare cohort described below to evaluate their transportability to a population outside of the VA healthcare system. This strict external validation was conducted separately from subsequent population-specific adaptation and model redevelopment analyses. The adaptation and redevelopment procedures are described in Section E.

### B. External validation data and cohort construction

For external validation, we used data from the SEER-Medicare linked database, including MDS assessments collected from nursing home residents between 2006 and 2018, and Medicare administrative claims, with cancer registry information obtained through linkage to the SEER cancer registry. The dataset included assessments recorded under both MDS version 2.0 and version 3.0. MDS 3.0, which became mandatory in 2010, introduced structural changes to the assessment instrument. To maintain consistency with the original CBIT framework, relevant assessment items from the MDS were mapped to the corresponding Barthel Index domains using the MDS-to-Barthel mapping framework reported by Wojtusiak et al. [1]. Because MDS 2.0 and MDS 3.0 use different response structures for some functional items, MDS 3.0 responses were additionally harmonized with corresponding MDS 2.0 functional-assistance levels before being assigned to Barthel Index scoring categories. Detailed variable definitions and mapping rules are provided in Table 11 and S2 Table.

**Table 11:** Example of scoring categories and mapping for the “Bladder Control” ADL.

	MDS 2.0	MDS 3.0	Barthel Index	Mapping
**Bladder control**	0 – continent	0 – always continent	0 – incontinent	MDS ‘0’ to Barthel ‘10’
	1 – usually continent	1 – occasionally incontinent*	5 – occasional accident	MDS ‘1’ to Barthel ‘5’
	2 – occasionally incontinent	2 – frequently incontinent	10 - continent	MDS ‘2’ to Barthel ‘5’
	3 – frequently incontinent	3 – always incontinent		MDS ‘3’ to Barthel ‘0’
	4 - incontinent			MDS ‘4’ to Barthel ‘0’

* Note: MDS 3.0 “1 – occasionally incontinent” was harmonized with MDS 2.0 “2 – occasionally incontinent” before assignment to the corresponding Barthel Index category.

Access to the data was obtained on March 8, 2022, under an approved Data Use Agreement. Access was granted through an application submitted by George Mason University to the SEER Program. SEER-Medicare is a limited dataset that includes Medicare beneficiaries diagnosed with cancer in SEER registry as well as a randomly selected sample of beneficiaries without cancer in those areas. Because the dataset combines beneficiaries with SEER-identified cancer cases with a 5% sample of beneficiaries without SEER cancer diagnoses, the resulting distribution is not nationally representative of the Medicare population [18]. The Institutional Review Board (IRB) determined that this project is exempt from IRB review according to federal regulations.

Cancer status was defined using SEER registry data. Patients were classified as having cancer if they had a SEER-confirmed diagnosis of at least one of the five cancer types included in this study: breast, lung, pancreas, prostate, and/or stomach cancer. Patients without a SEER-confirmed diagnosis of any of these five cancer types were classified as non-cancer. Cancer and non-cancer classifications were assigned at the patient level based on SEER registry status and were subsequently summarized across available MDS assessments for cohort description and subgroup analyses.

The original CBIT framework excluded patients without sufficient longitudinal MDS information to model functional change over time. Consistent with this longitudinal validation framework, we required patients to have sufficient longitudinal MDS follow-up for temporal outcome ascertainment and model evaluation. Specifically, patients were included if they had at least three consecutive years of MDS assessments. This criterion may preferentially select long-stay nursing home residents with greater longitudinal observation and may therefore limit generalizability to shorter-stay residents.

The final validation cohort included 843,836 nursing home residents identified through the SEER-Medicare linked data, contributing 8,257,141 MDS assessments. This cohort enabled evaluation of CBIT transportability in a large nursing home population outside of the VA healthcare system. Fig 3 presents a flow diagram summarizing the cohort selection process.

**Fig 3:**
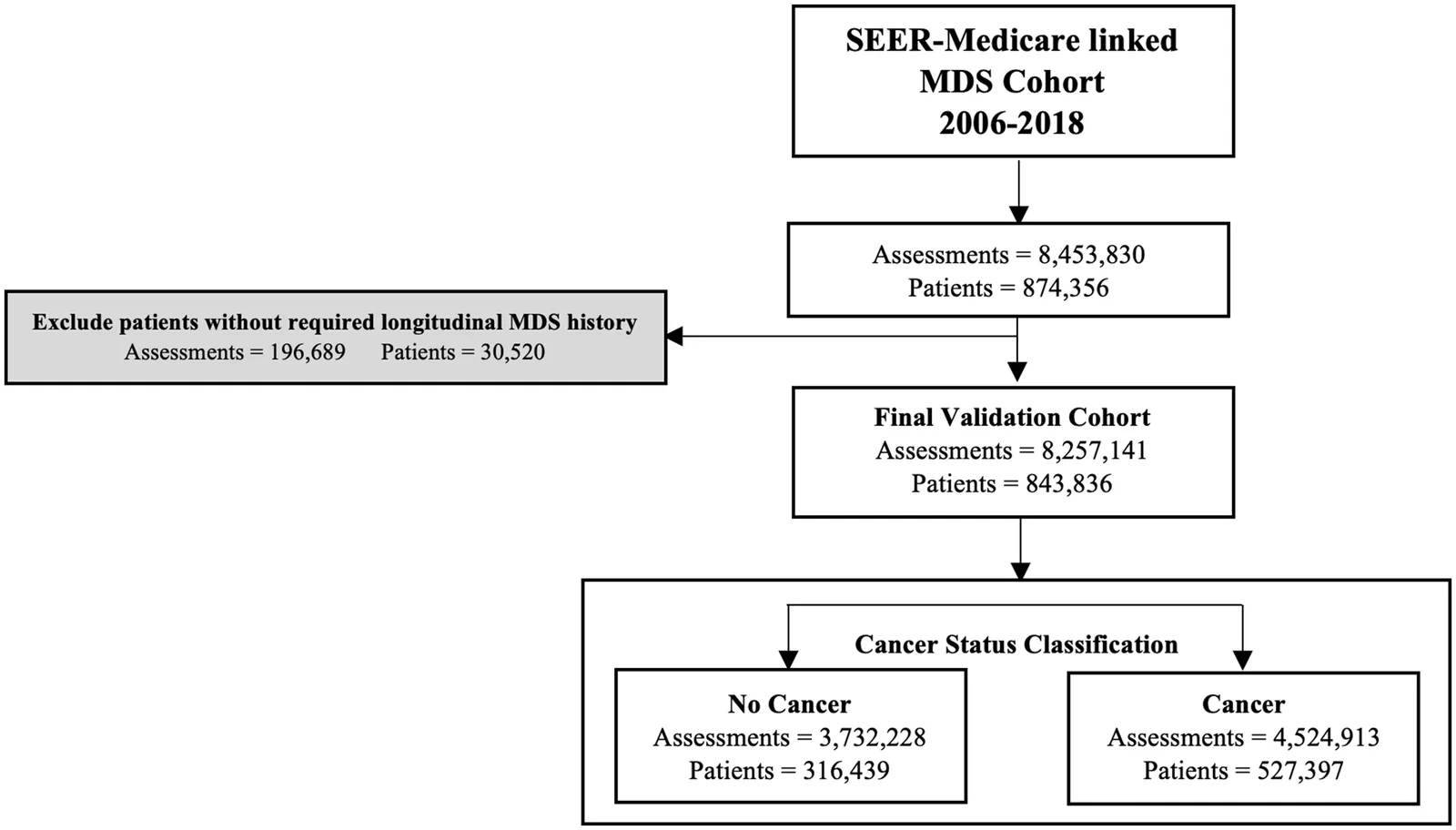
Flowchart illustrating the cohort selection process.

### C. Outcome and predictor variables

Outcome variables were defined to match those used in the original CBIT framework and consisted of nine measures representing functional status in Activities of Daily Living (ADLs) based on the Barthel Index: bathing, bladder control, bowel continence, dressing, eating, grooming, toileting (toilet use), transferring (transfers), and walking (mobility). Although the underlying Barthel Index measures are scored categorically, the original CBIT framework transformed each ADL into a binary outcome representing full independence versus any level of dependency [1]. The same binary outcome definition was used in the present study to maintain consistency with the pretrained CBIT models.

The original CBIT framework was developed using MDS 2.0 assessments, with functional assessment items operationalized into the nine Barthel Index ADL domains. The Barthel Index scoring categories used in CBIT were based on the scoring scheme reported in the original CBIT supplementary material [1], which reproduced the standard Barthel Index scoring framework described by Wojtusiak et al. [20]. Because the present external validation cohort contained both MDS 2.0 and MDS 3.0 assessments, MDS 3.0 functional assessment items were harmonized to the corresponding MDS 2.0 functional-assistance levels and subsequently mapped to the same Barthel Index scoring categories used by CBIT. This harmonization was necessary because MDS 3.0 introduced changes in the response structure and wording of several functional assessment items. The resulting MDS-derived measures were then converted to the binary dependency outcomes required by the pretrained CBIT models. Table 11 provides an example of the scoring and mapping procedure for bladder control, and the complete MDS 2.0 and MDS 3.0 harmonization rules are provided in S2 Table.

For MDS 3.0, harmonization was based on correspondence in the level of functional assistance represented by the response categories rather than direct equivalence of the numeric MDS response codes. Thus, MDS 3.0 categories were aligned with the corresponding MDS 2.0 functional-assistance levels before assignment to the CBIT Barthel Index scoring categories. The resulting measures represent operationalized Barthel Index categories derived from MDS assessments and should not be interpreted as identical to directly administered Barthel Index assessments.

For each prediction timeframe, the MDS assessment serving as the index assessment defined the prediction date. For current predictions (τ = 0), the ADL status recorded at the index assessment was used as the outcome. For future predictions, outcomes were defined at the same prediction timepoints used in the original CBIT framework: 90 days (3 months), 180 days (6 months), and 365 days (12 months) after the prediction date [1]. Following the data structure of the original CBIT framework, each MDS assessment was treated as an observation.

For each prediction timeframe, when multiple MDS assessments were available, the first assessment was used to define the outcome. If no assessment was available at a given prediction timeframe, the observation did not contribute an outcome for that timeframe. Thus, an observation could contribute to one prediction timeframe but not another depending on the availability of an MDS assessment for that timeframe. Patients who were discharged or died before an assessment therefore did not contribute an outcome for that prediction timeframe. Future outcomes were based only on observed MDS assessments available at the corresponding prediction timeframe.

Predictor variables were reconstructed to match the original CBIT model. These included demographic characteristics (age as a continuous variable and sex and race as categorical variables) and clinical history derived from diagnosis codes. Diagnoses recorded using ICD-9-CM and ICD-10-CM codes were mapped to the 281 Clinical Classifications Software (CCS) categories used in the original CBIT framework [1].

For each CCS condition, two temporal variables were generated relative to the prediction date to capture diagnosis recency and chronicity: *ccs*_i_^max^, representing the number of days since the first recorded diagnosis, and *ccs*_i_^min^, representing the number of days since the most recent diagnosis. Only diagnosis information available on or before the prediction date was used to construct these predictors. Conditions absent from a patient’s clinical history were encoded using sentinel values (-999,999 for *ccs*_i_^max^ and 999,999 for *ccs*_i_^min^), consistent with the original CBIT model specification. These values indicated that no corresponding diagnosis was present in the available clinical history and did not represent observed diagnosis dates. No imputation was performed for sex or race because these variables had no missing values. Missing age values were imputed using the mean age.

For re-evaluation models, the most recently available functional assessment preceding the prediction date was additionally included as predictor information, consistent with the original CBIT framework. For evaluation models, prior functional assessment information was not included.

Separate analytic datasets were constructed for prediction at four timepoints: current status (τ = 0), 3 months (τ = 90 days), 6 months (τ = 180 days), and 12 months (τ = 365 days). Predictor information was restricted to data available on or before the prediction date, while subsequent MDS assessments were used only for outcome ascertainment. This temporal separation was maintained to prevent information from after the prediction date from entering the predictor set.

### D. Model evaluation, subgroup performance, and calibration

All 72 pretrained CBIT models, including evaluation (*M*_*E*_^*d*^_*τ*_) and re-evaluation (*M*_*RE*_^*d*^) models, were applied directly to the reconstructed SEER-Medicare datasets without retraining, recalibration, or modification of model parameters. The pretrained models were evaluated independently of the original VA development data to assess their transportability to the SEER-Medicare nursing home population.

#### Overall model performance.

Model discrimination was primarily assessed using Area Under the Receiver Operating Characteristic Curve (AUC). AUC measures the ability of the model to distinguish between observations classified as independent and those classified as having functional dependency for each ADL outcome. AUC was calculated separately for each of the nine ADL outcomes and at each prediction timeframe: current status (τ = 0), 3 months (τ = 90 days), 6 months (τ = 180 days), and 12 months (τ = 365 days).

The binary ADL outcomes used for evaluation were derived from the corresponding Barthel Index scores using the outcome thresholds defined in the original CBIT framework. An ADL was classified as dependent when its Barthel Index score was below the domain-specific independence threshold. The thresholds were 10 for eating, toileting, dressing, bowel continence, and bladder control; 5 for grooming and bathing; and 15 for transferring and walking.

Additional classification metrics, including accuracy, precision, and recall, were calculated for each ADL outcome and prediction timeframe. For these threshold-dependent metrics, predicted probabilities were converted to binary predictions using a probability threshold of 0.50, with probabilities greater than or equal to 0.50 classified as predicted dependency. Accuracy represents the proportion of correctly classified observations, precision represents the proportion of predicted dependent observations that were correctly classified as dependent, and recall (sensitivity) represents the proportion of truly dependent observations correctly identified by the model. In contrast, AUC was calculated directly from the continuous predicted probabilities and was therefore independent of a classification threshold.

For summary tables reporting mean ± standard deviation, the mean represents the average performance estimate across the nine ADL outcomes within each model type and prediction timeframe. The corresponding standard deviation represents variation in the performance estimates across the nine ADLs. Thus, these summary statistics describe between-ADL variation rather than variation across repeated samples or patients.

#### Subgroup performance and generalizability.

To assess whether model discrimination was consistent across clinically and demographically distinct populations, subgroup analyses were performed by age group, sex, race/ethnicity, cancer status, and cancer type. For each subgroup, AUC was calculated separately for each ADL outcome and prediction timeframe using the same pretrained model predictions and outcome definitions as in the overall analysis.

Cancer status and cancer type were defined using SEER registry information as described in Section B. These analyses were intended to characterize variation in predictive discrimination across subgroups and to evaluate the generalizability of pretrained models across the heterogeneous external population. Because subgroup analyses were based on AUC, they were interpreted as assessments of differences in model discrimination rather than as comprehensive measures of algorithmic fairness.

#### Calibration assessment.

Calibration was evaluated to assess the agreement between predicted probabilities and observed outcome frequencies in the external population. Three complementary measures were used. First, calibration curves were generated by comparing predicted probabilities with observed outcome frequencies across probability groups. Second, expected calibration error (ECE) was calculated as the weighted average of the absolute difference between predicted probabilities and observed outcome frequencies across probability groups. Third, the Brier score was calculated as the mean squared difference between predicted probabilities and the observed binary outcomes, with lower values indicating better overall probabilistic accuracy.

Calibration was assessed separately for each model type, ADL outcome, and prediction timeframe to characterize changes in calibration as the prediction horizon increased.

### E. Model adaptation using external-domain data

To evaluate whether population-specific adaptation of the pretrained CBIT models to the external nursing home SEER-Medicare population could improve predictive performance, the pretrained CBIT models were incrementally adapted using subsets of the external nursing home SEER-Medicare population. This adaptation analysis was performed separately from the strict external validation described above. The original predictor structure and outcome definitions were retained to preserve consistency with the original CBIT framework, while the model parameters were adapted using the external domain training data.

The external nursing home SEER-Medicare cohort was randomly divided at the patient level into training and testing sets, with 80% of patients assigned to the adaptation training set and 20% reserved as a held-out test set. Patient-level splitting ensured that observations from the same patient were not present in both the training and testing sets. The held-out test set remained unchanged across all adaptation analyses and was not used for model fitting or adaptation.

For each ADL outcome and prediction timeframe, the corresponding CBIT model was adapted using external nursing home SEER-Medicare population training data with the same predictor variables and binary outcome definitions used in the external validation analysis. The adapted models were evaluated on the held-out 20% patient-level test set using ROC AUC calculated from predicted probabilities. Thus, the performance of the adapted models reflected their ability to discriminate functional dependency among the external nursing home SEER-Medicare patients who were not included in model adaptation.

To evaluate the amount of external-domain data required for adaptation, models were adapted using progressively larger proportions of the SEER-Medicare adaptation training dataset. Training data proportions of 0.01%, 0.1%, 1%, 10%, 20%, 30%, 40%, and 50% were examined. For each proportion, the corresponding model was adapted using the specified subset of the adaptation training data and evaluated on the same held-out test set. This analysis characterized how predictive performance changed as the amount of external domain data available for model adaptation increased.

As a comparator to population-specific adaptation, fully redeveloped models were also evaluated using the external nursing home SEER-Medicare population adaptation training data. Redeveloped models used the same predictor variables, ADL outcome definitions, and prediction timeframes as the corresponding CBIT models but were fitted independently using the external nursing home SEER-Medicare population data without relying on the pretrained CBIT model parameters. The redeveloped models were evaluated on the same held-out 20% patient-level test set used for the adaptation analyses. This comparison was used to assess whether adapting the pretrained models provided performance comparable to full model redevelopment when external-domain data were available.

Fig 4 and Fig 5 summarize the overall study workflow and the model adaptation framework, respectively.

**Fig 4:**
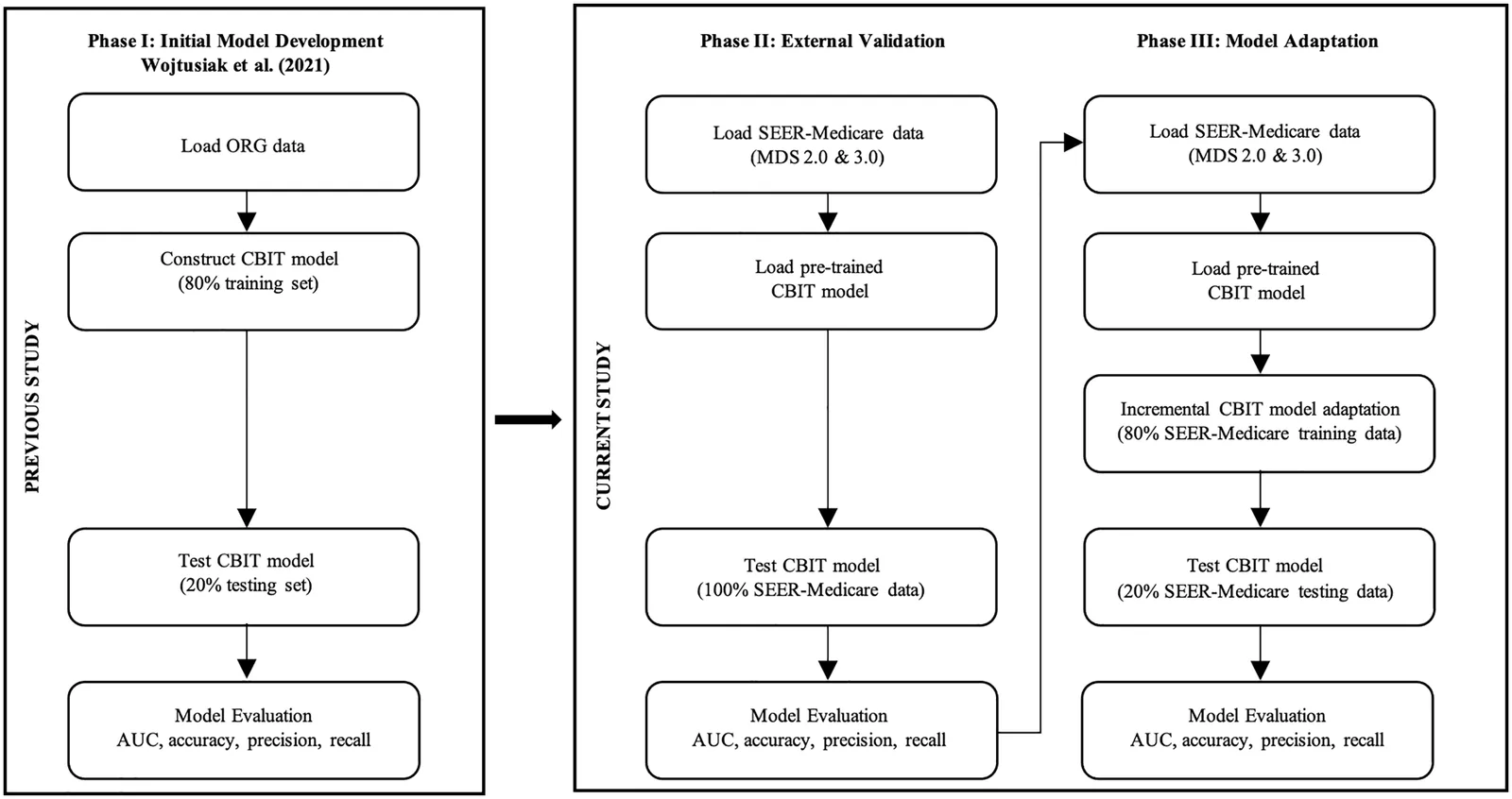
Flowchart illustrating the study design overview.

**Fig 5:**
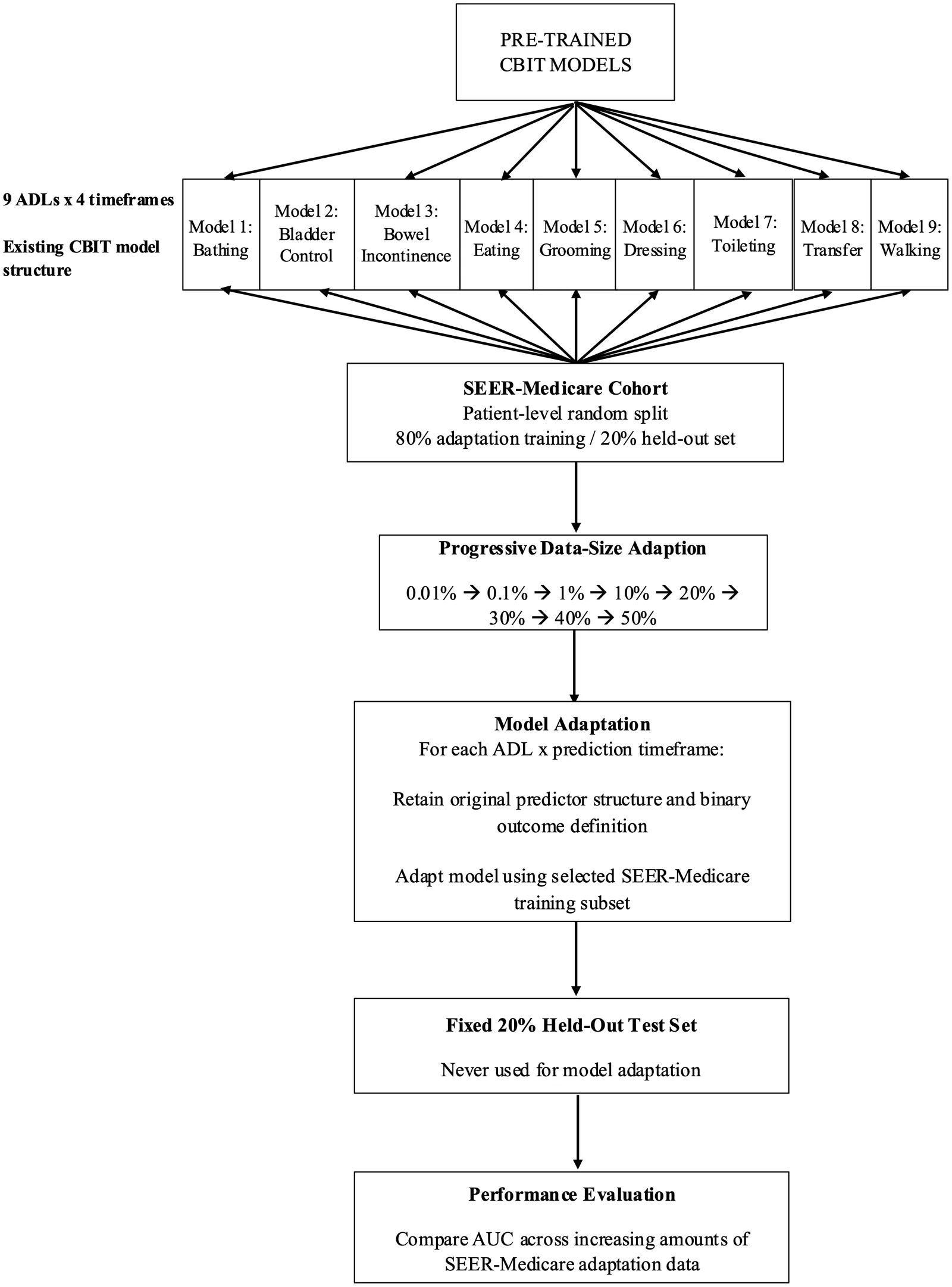
Flowchart illustrating the model adaptation process.

## Conclusion

Overall, this study demonstrates that transporting a pretrained clinical ML model involves more than determining whether its original performance is preserved in a new population. CBIT experienced meaningful performance degradation under strict external validation, particularly for longer-term prediction and for evaluation models without prior functional status. However, population-specific adaptation substantially recovered discrimination, and performance generally improved with increasing amounts of local data. Comparison with SEER-Medicare model reconstruction suggests that adaptation may provide a practical alternative to full model reconstruction or redevelopment when local data or development resources are limited, whereas full reconstruction or redevelopment remains an option when sufficient data and resources are available. More broadly, these findings support a structured approach to clinical ML transportability in which external validation is followed, when necessary, by population-specific adaptation and empirical comparison with model reconstruction or redevelopment.

### Ethics approval and consent to participate

The study was deemed non-human subject research. The Institutional Review Board (IRB) Office determined that this project is exempt from IRB review according to federal regulations.

## Supporting information

S1 Table ADL-specific performance results for evaluation and re-evaluation models across prediction timeframes. (XLSX)

S2 Table MDS Harmonization to Barthel Index Categories. (PDF)

S1 Fig ADL-specific calibration plots for evaluation and re-evaluation models across prediction timeframes. (XLSX)

S2 Fig ADL-specific model adaptation graphs across varying adaptation dataset sizes for evaluation and re-evaluation models. (XLSX)
