# Supplementary material for: External validation and adaptation of the Computational Barthel Index Tool in a large nursing home cohort
Source: PLOS Digit Health. 2026 Oct 23;5(10):e0001793. doi: 10.1371/journal.pdig.0001793 (PMC13693907; doi:10.1371/journal.pdig.0001793)
Supplement: Supplementary file 1 [file pdig.0001793.s002.pdf]

S2 Table. MDS 2.0 and MDS 3.0 Harmonization to Barthel Index Categories

	MDS 2.0	MDS 3.0	Barthel Index	Mapping
**Bathing**	0 – independent<br>1 – supervision<br>2 – physical help limited to transfer<br>3 – physical help in part of bathing<br>4 – total dependence	0 – independent<br>1 – supervision<br>2 – physical help limited to transfer<br>3 – physical help in part of bathing<br>4 – total dependence	0 – dependent<br>5 – independent	MDS ‘0’ to Barthel ‘5’<br>MDS ‘1’ to Barthel ‘0’<br>MDS ‘2’ to Barthel ‘0’<br>MDS ‘3’ to Barthel ‘0’<br>MDS ‘4’ to Barthel ‘0’
**Bladder Control**	0 – continent<br>1 – usually continent<br>2 – occasionally incontinent<br>3 – frequently incontinent<br>4 – incontinent	0 – always continent<br>1 – occasionally incontinent*<br>2 – frequently incontinent<br>3 – always incontinent	0 – incontinent<br>5 – occasional accident<br>10 – continent	MDS ‘0’ to Barthel ‘10’<br>MDS ‘1’ to Barthel ‘5’<br>MDS ‘2’ to Barthel ‘5’<br>MDS ‘3’ to Barthel ‘0’<br>MDS ‘4’ to Barthel ‘0’
**Bowel Incontinence**	0 – continent<br>1 – usually continent<br>2 – occasionally incontinent<br>3 – frequently incontinent<br>4 – incontinent	0 – always continent<br>1 – occasionally incontinent*<br>2 – frequently incontinent<br>3 – always incontinent	0 – incontinent<br>5 – occasional accident<br>10 – continent	MDS ‘0’ to Barthel ‘10’<br>MDS ‘1’ to Barthel ‘5’<br>MDS ‘2’ to Barthel ‘5’<br>MDS ‘3’ to Barthel ‘0’<br>MDS ‘4’ to Barthel ‘0’
**Dressing**	0 – independent<br>1 – supervision<br>2 – limited assistance<br>3 – extensive assistance<br>4 – total dependence	0 – independent<br>1 – supervision<br>2 – limited assistance<br>3 – extensive assistance<br>4 – total dependence	0 – dependent<br>5 – needs help<br>10 – independent	MDS ‘0’ to Barthel ‘10’<br>MDS ‘1’ to Barthel ‘5’<br>MDS ‘2’ to Barthel ‘5’<br>MDS ‘3’ to Barthel ‘0’<br>MDS ‘4’ to Barthel ‘0’
**Eating (Feeding)**	0 – independent<br>1 – supervision<br>2 – limited assistance<br>3 – extensive assistance<br>4 – total dependence	0 – independent<br>1 – supervision<br>2 – limited assistance<br>3 – extensive assistance<br>4 – total dependence	0 – unable<br>5 – need helps<br>10 – independent	MDS ‘0’ to Barthel ‘10’<br>MDS ‘1’ to Barthel ‘5’<br>MDS ‘2’ to Barthel ‘5’<br>MDS ‘3’ to Barthel ‘0’<br>MDS ‘4’ to Barthel ‘0’
**Grooming**	0 – independent<br>1 – supervision<br>2 – limited assistance<br>3 – extensive assistance<br>4 – total dependence	0 – independent<br>1 – supervision<br>2 – limited assistance<br>3 – extensive assistance<br>4 – total dependence	0 – needs help<br>5 – independent	MDS ‘0’ to Barthel ‘5’<br>MDS ‘1’ to Barthel ‘0’<br>MDS ‘2’ to Barthel ‘0’<br>MDS ‘3’ to Barthel ‘0’<br>MDS ‘4’ to Barthel ‘0’
**Toileting (Toilet use)**	0 – independent<br>1 – supervision<br>2 – limited assistance<br>3 – extensive assistance<br>4 – total dependence	0 – independent<br>1 – supervision<br>2 – limited assistance<br>3 – extensive assistance<br>4 – total dependence	0 – dependent<br>5 – needs help<br>10 – independent	MDS ‘0’ to Barthel ‘10’<br>MDS ‘1’ to Barthel ‘5’<br>MDS ‘2’ to Barthel ‘5’<br>MDS ‘3’ to Barthel ‘0’<br>MDS ‘4’ to Barthel ‘0’
**Transferring (Transfers)**	0 – independent<br>1 – supervision<br>2 – limited assistance<br>3 – extensive assistance<br>4 – total dependence	0 – independent<br>1 – supervision<br>2 – limited assistance<br>3 – extensive assistance<br>4 – total dependence	0 – unable<br>5 – major help<br>10 – minor help<br>15 – independent	MDS ‘0’ to Barthel ‘15’<br>MDS ‘1’ to Barthel ‘10’<br>MDS ‘2’ to Barthel ‘10’<br>MDS ‘3’ to Barthel ‘5’<br>MDS ‘4’ to Barthel ‘0’
**Walking**	0 – independent<br>1 – supervision<br>2 – limited assistance<br>3 – extensive assistance<br>4 – total dependence	0 – independent<br>1 – supervision<br>2 – limited assistance<br>3 – extensive assistance<br>4 – total dependence	0 – immobile 5 – wheelchair independent<br>10 – walks with help<br>15 – independent	MDS ‘0’ to Barthel ‘15’<br>MDS ‘1’ to Barthel ‘10’<br>MDS ‘2’ to Barthel ‘10’<br>MDS ‘3’ to Barthel ‘5’<br>MDS ‘4’ to Barthel ‘0’

* **Note:** Barthel Index scoring categories were based on the scoring scheme reported in the original CBIT supplementary material [1]. Because MDS 2.0 and MDS 3.0 use different response structures for some functional items, MDS 3.0 response categories were harmonized with the corresponding MDS 2.0 functional-assistance levels before assignment to the CBIT Barthel Index scoring categories. For bladder and bowel control, the MDS 3.0 category “1 – occasionally incontinent” was aligned with the corresponding MDS 2.0 “2 – occasionally incontinent” category. The resulting measures represent operationalized Barthel Index categories derived from MDS assessments and should not be interpreted as identical to directly administered Barthel Index assessments.
